# Letter from Dr. Westcott

**Published:** 1846-06

**Authors:** A. Westcott


### ARTICLE VI.

### *Letter from* DR. WESTCOTT.

IN giving place to Dr. Bakers's communication in the Journal, we thought it no more than just, that our collegue Dr. Westcott, should see it, previously to its publication, as it related principally to him. We, therefore, sent it to Dr. W., and have received, in reply to our note which accompanied Dr. B's communication, the following letter.—*Balt. Ed.*

SYRACUSE, *May* 12, 1846.

FRIEND HARRIS:

Your favor enclosing Dr. E. Baker's article, or counter "protest," for my inspection and review, was received by yesterday's mail. Having just transmitted to you, for publication a review of a very similar document from C. C. Allen, M. D., I do not regard it worth while to encumber the pages of the Journal with any separate review of Dr. Baker's article. I have given it a very careful reading and think it reflects upon the author much more credit for literature, than for sound views, in regard to the art of stopping teeth. I am certainly very glad to have Dr. Baker speak for himself, and I am, on the whole, pleased with the style and temper of his remarks, notwithstanding, he takes me to task for impertinence. But in regard to his views of amalgams, for stopping teeth, and also upon several other topics which he brings up, I of course hold a very different opinion from those set forth by him, in his communication. Although I have taken some pains to understand the precise aim of this production, yet I confess, it is in my mind, somewhat enigmatical. Whether the object of his communication is to defend the claims of amalgams, and the rights of their advocates, or to demonstrate that those who have hitherto considered them as unfit for filling teeth, and who *have not changed their minds*, are "fools ;" or to prove that the persecution which the doctor claims for himself and friends, necessarily rank him and them, with the sages and martyrs of former times, who also suffered, because they were in advance of their age, in wisdom and goodness ; or whether it was more to get a medium, through which to administer to me, a friendly castigation, is not quite certain. But perhaps these may not be considered important queries.

In regard to that part of his article intended expressly for my benefit, I have only to say, that I trust my *juniority*, which he takes so much pains, by frequent repetition, to hold out in bold relief, will enable me to out grow the cicatrices, with which his lashes might otherwise mark me for life. You doubtless noticed that his position upon most of the topics which he has discussed in common with Dr. Allen, are the same, or at least very similar to those taken by the latter. For example, both contend that amalgams may be used judiciously in certain cases, yet neither of them has seen fit to inform the profession, what we are to understand by this phrase, "certain cases." Now it occurs to me, that if Dr. Baker really thinks amalgam "the very best filling in certain cases," and is, at the same time, as he would have us understand, far more anxious to "*assist* mankind, than to be

*commended*," that his benevolence and philanthropy would have suggested to him the propriety of informing the profession, *how* this great assistance could be rendered to mankind. Perhaps he thinks this obligation has been discharged in the expression of his views upon this point to the *Society*, at the last meeting, and which were recorded by Dr. E. Parmly, in a note appended to his introductory address.

Dr. Parmly's record, (Vol. vi. No. 1, of the Journal,) is as follows—"He" (Dr. Baker,) "admitted before you all, that it is a *bad filling*—that it is *the worst kind of filling*—that it is *a nasty* filling, and that it is *the worst thing in the world to fill teeth with, except as a filling for the mere shell of a tooth, that will bear nothing else.*" In relation to the sentiments as above expressed, Dr. P. observes in the same note, that "such an admission from such a man as Dr. Baker, should make the face of every honorable man blush, to own that he uses it, or to give the slightest encouragement, or in any way sanction its use."

Now if such is the description of the doctor's "certain cases," and he has made no other revelation to my knowledge, I fear he will be puzzled to make it appear that a filling, which in *small* cavities, is a "bad" and "nasty" filling, is any other than such in large, or as would appear from his own description, *monstrous* cavities.

It is a logic which *common* minds cannot comprehend. Now in respect to filling teeth, I am willing to take the position, and will endeavor to sustain it by *works*, that any tooth which can be materially benefitted by any filling, can be stopped with gold or tin.

To put this question to a practical test, I would like to have Dr. B. present to the society, at the next meeting, some cases in which he thinks amalgams should be used, and if it is decided by the society, or by a committee appointed by them, that the teeth so presented, are proper cases for filling in any way, or with any material, I will volunteer to make the attempt with *gold*. These cases might be presented from year to year, and the work examined. In this way one question at least might be settled, viz. whether mineral paste is used by persons competent to do *good* work, as a matter of *convenience*, or as a matter of *necessity*. Even this would be placing the issue on the ground, that the "paste" possessed no positively noxious qualities, which is a supposition by no means to be admitted, after the abundant proof which has been exhibited to the contrary.

The society viewing the subject in this light, entered an unconditional protest against it. That Dr. B. might have been surprised at the views of the society, is highly probable, from his last communication, but is it not strange that he was surprised to find the pledge, when it came to his hand, "in accordance with the resolutions," which prescribed its particular form?—especially when you know that he was present during the entire discussion of these resolutions, and when (if I mistake not) they were passed? Not only so, but I boldly assert that he consented to acquiesce in this very proceeding of the society.

The constitutionality of this proceeding, the doctor discusses somewhat learnedly, quoting (with slight alteration) from Webster's large Dictionary, the definition and application of the term, constitution. His reasoning under this head is all very well. I freely admit, that if this act can be shown to conflict either with the true intent of the constitution, or with the legitimate objects of the association, that it is null and void, but till then, it will stand. *Perhaps*, "striking from the roll," does not mean "expelling," but there can be no doubt that it would at least mean *suspending*, and the act is, therefore, if it has any virtue, one which would disfranchise every member, whose name was so stricken from the roll, and to restore such members, it would require direct action of the society, or of those members in full standing. Now, if deciding questions by ballot, is the "*brute force*," which Dr. B. anticipates in his communication, that will be exercised, then I think he will not be disappointed, for I have no idea that the legitimate voters in the society, will see fit to revoke their decision, made at the last meeting, however brutal it may be regarded, by those who find it convenient to use amalgams.

Although I have protracted my letter to much greater length than I anticipated doing when I commenced it, yet I cannot refrain from noticing two or three other points, upon which Dr. B. lays some stress.

The doctor refers to the resolutions of the Virginia Society, to show the tyranny and brutality of the measure of the American Society, and seems to rely upon them for sympathy. But how, entertaining such views of the *practice*, as he does, can he expect sympathy and support from a society who have declared, by a resolution unanimously passed, and without the least qualification, the very article for which Dr. B. contends to be, "*entirely unfit for, and highly objectionable as fillings for carious teeth*," and "*that the* USE *of amalgams in dental practice, is* EMPIRICAL," and who, moreover, in the same resolution, that "*it is hereby declared to be* MALPRACTICE?"

Now, how a man who USES and declares his determination to use this very same "*nasty*" material, which is in the above quoted resolution of the Virginia Society, so fully and so uncompromisingly denounced and proscribed, can look to this source for countenance or authority, I have not the sagacity to comprehend! True, this society complain of the *means* employed by the American Society, to rid herself of this blighting evil, which both societies view in the same light, yet the Virginia Society have, by no means extended either sympathy or encouragement, to any who are *known* to USE this article, which they so strongly denounce, much less to those who publicly declare their intention to continue its use.

Again, Dr. Baker refers to C. S. Brewster, as a *foreign "prophet*," and who for *this* reason, should be heard and accredited. Now, if Dr. B. thinks Mr. Brewster's authority enhanced by his residence in Paris, I beg leave to differ with him in opinion. That Mr. Brewster has *used* the amalgam for sometime, is to me no new fact, but I confess, I had never dreamed that this constituted any reason why we, who live in the "land of steady habits," and who are in most respects, very differently situated, should adopt either his sentiments or practice. Nay, on the other hand, I had gone upon the supposition that the very striking change which had been wrought in Mr. Brewster's views and practice, had arisen somewhat from his particular residence, and the peculiar circumstances, by which he has been surrounded for the last few years. To say the least, as I view them, they have been such as are well calculated to enhance all the virtues any practice does possess, which makes so little tax upon time, and physical exertion and skill, as does the practice in question. It strikes me, therefore, that if a residence in Paris can have any bearing upon Mr. Brewster's testimony as authority upon *this* subject, that it would tend to weaken, rather than strengthen it, and I think it not unfair to presume, that had this accomplished, and highly reputed gentleman remained an *American* dentist, that he would still be found "thundering forth universal condemnation against the use of this article." In relation to Mr. Brewster's testimony, relative to the *universal* employment of amalgam for stopping teeth, by European dentists, it by no means accords with my understanding of this matter. But my time will not now permit me to enlarge upon this point. At a proper time, I will lay before the readers of the Journal, some statistics which I think will somewhat change the complexion of this feature in the case.

Before I close my letter, I wish to notice briefly, Dr. Baker's reprimand for my allusion to *him* on a former occasion. He observes in relation to my remarks, that they are "objectionable, both in *manner*, *matter* and statement of facts."

You being familiar with the history of the transactions which gave rise to the article, to which Dr. B. alludes, will readily understand the following statement of circumstances which will serve to account for, if not excuse, any allusion which I made to Dr. B., in the article of which he complains. These circumstances were as follows:

The subject of amalgams had undergone a long discussion at the late annual meeting of the society, and as was supposed by all, fully disposed of. Dr. Baker was present during the whole session, and participated in the discussion, and notwithstanding he at first objected to signing the pledge, *described in the resolutions*, then passed, he *did* consent to acquiesce in the measure, which of course included signing said protest. The meeting broke up with this understanding; but no sooner was the pledge issued, than he addressed a letter to me, showing most plainly, that he had been "convinced against his will." Hence, held his *old* "opinion still." More than this—he, in this letter, denounced the proceedings of the society, in the strongest language, and in terms illy becoming one situated as he was, after having acquiesced in these proceedings, and accepted an *office*, *after* the resolutions were passed, the protest ordered, and *explicitly described.*

About this time, I received several other letters of similar import, but not from any other man, who was present during the meeting, at which the matter had been discussed. To these several attacks upon the American Society, from her own members, and especially the one from Dr. Baker, I thought some reply was called for. Now, who was to make this reply, the society was, pro tem., virtually disbanded, and had every member taken the same course, as did Dr. B., it would have resulted in a *final* disbanding of the association, for all were alike suspended, till they had complied with the requisition, or had signed and returned the circular. Now, it struck me as highly proper, that the society should be apprised of the exact standing of the matter, at this juncture. So much importance did I attach to this new and unexpected turn, that I consulted the President in relation to the propriety of calling an extra session, to make some provision for this unlooked for exigency. It is very evident that no one but myself could make any report of progress, as the whole business of issuing and receiving the protests, and all other documents pertaining thereunto, was put into my hands. I therefore, as the society's agent, through the Journal, as their organ, made the report contained in the article of which Dr. B. complains. I made just so much allusion to Dr. B's letter as I thought the case required, considering him the *leader* of his party. So much for the occasion and the "*manner*." I leave it to the society, as whose agent I was acting, to declare whether my course was justifiable, or whether, as Dr. B. claims, it was reprehensible.

As to the "matter and statement of facts," which he makes also a subject of complaint, I have only to say that I did not intend by any means to misrepresent him. But on receiving a letter from him, a short time after my article appeared in the Journal, charging me with "gross misstatements," &c., I felt that I owed it to myself, as well as to him, to see if some retraction should not be made, or whether it was not possible I had misunderstood, and hence misstated in respect to some of the points of complaint.

To this end, I made a very thorough investigation in regard to every point involved, and the result has confirmed the belief, that I have nothing to retract, not even excepting my allusion to the Mallans persecution.* True, Dr. Baker's name was not on the card which was circulated, to expose their villainy, nor was he on the committee which issued it, but the doctor will not deny that he was privy to, and encouraged that measure, and paid *liberally* towards footing the bills incurred by prosecuting it, all of which I regard very creditable to him—creditable alike, to his wish to do good, and his liberality.

I will only notice one other portion of Dr. B's communication. I allude to his effort to rank those who use amalgams "in certain cases," and *are persecuted therefor*, with the benefactors and wise men of former times, who suffered similar persecution.

He has brought forward, a host of "pictorials and illustrations," to show that great men have *always* been persecuted. True he does not add in words—hence we are persecuted—or this is just our case, but it would verily appear, that his reasoning invites us to fill up the blank with one of these expressions. It appears to me, however, that the doctor would not relish all the applications that might be made of his argument. The Crawcours were persecuted as we learn, from Dr. B. himself, and *I have no doubt* that the Mallans were also. Now this logic, without limit, would apply as well to them, as to the greatest benefactor that ever lived, and they might with the same plausibility claim, that they were persecuted, because the world was too ignorant to appreciate their real merit and character. The truth is, that persecution can of itself, prove nothing more than that the sentiments or acts of those persecuted, are at variance with the views of the persecutors. It would not prove either

* Notwithstanding, I still regard Dr. Baker as one who was engaged in this persecution, if it can be so styled, yet perhaps it cannot be justly said, that he was *"foremost"* in it, as I have no evidence that he originated the scheme, or that he was the leader in carrying it on. This expression in my original article, rather alluded to the fact, that "he paid more money than any other individual," to make the measure efficient.

party to be made up either of wise men or fools, and hence I am unable to see the bearing of his logic, as applied to the persecution of which he complains. You will please pardon me for the great length of this letter, and believe me,

Cordially, yours,

A. WESTCOTT.

